# Supplementary material for: Are carbon emissions trading and green financial instruments synergistic? -Comprehensive quantitative research based on content analysis
Source: PLoS One. 2024 Mar 7;19(3):e0298601. doi: 10.1371/journal.pone.0298601 (PMC10919601; doi:10.1371/journal.pone.0298601)
Supplement: S1 Data — (ZIP) [file pone.0298601.s001.zip › Data/地级市Z_F工作报告.zip!/┴╔─■/▒╛╧¬╩╨/└┤╫╘╣1⁄2╓┌║┼í╛┬φ┐╦╩2╛▌═°í┐_2010.docx]

各位代表：

现在，我代表市人民政府向大会报告工作，请予审议，并请市政协委员和其他列席同志提出意见和建议。

一、2009年工作回顾

过去的一年，是很不寻常、极其不易的一年。面对国际金融危机的严重影响，在省委、省政府和中共本溪市委的正确领导下，全市各族人民深入学习实践科学发展观，牢牢把握“保增长、保民生、保稳定、促振兴”的工作大局，抢抓机遇，创新实干，共克时艰，经受住了本溪发展史上少见的严峻挑战和重大考验。预计全市生产总值实现680亿元，增长18.2%；地方财政一般预算收入50.3亿元，增长18.6%；全社会固定资产投资350亿元，增长56.2%；社会消费品零售总额167亿元，增长18%；城镇居民人均可支配收入14642元，增长10%；农民人均纯收入7058元，增长14.5%。全市主要经济指标增幅高于全省平均水平，高于我市振兴以来平均水平，全面完成了市十四届人大二次会议确定的各项任务，也为全省经济发展做出了积极贡献。

（一）全力以赴保增长，经济发展和结构调整取得新成果

保增长就是保企业，工业经济发挥了支撑作用。采取政策扶持、银企对接、协调保障、强化服务等措施，帮助企业渡难关。全市规模以上工业企业当年新增112户，总数达到663户；完成增加值351.6亿元，增长23.7%，高于全省平均增幅7.2个百分点。工业用电量持续增长，增幅排在全省第一位。本钢、北钢在应对金融危机过程中彰显了大企业的英雄本色。本钢产量增长20%、创历史最高水平，汽车板材销售增长50%，产品出口量在国内钢铁行业排第三位。北钢固定资产投资增长84%，工业总产值增长78%。两大企业为全市经济发展做出了重大贡献。县区规模以上工业增加值保持了30%的增幅。争取到国家和省工业技改贴息资金超亿元，支持了重点技改项目400个，当年竣工投产100个。历史上第一次走出去并购了海外科技型企业，引进海外研发团队7个。本溪首次拥有了“苍松”牌药品、“五女山”牌绿色食品和“龙宝”牌参茸3个全国驰名商标。全年开发新产品230项，实现销售收入300亿元。高新技术产业实现增加值72亿元，增长26.8%。非公有制经济完成增加值364亿元，增长26.1%。

扩内需增投资，交通能源基础设施建设突飞猛进。过去一年是本溪有史以来基础设施项目开工建设数量最多、档次水平最高、投入最大的一年，一大批交通能源类基础设施项目全面开工建设。彻底改变桓仁地区交通面貌的“两高一铁”项目全线开工。沈本城际轨道项目开始征地拆迁。沈本产业大道本溪段基本竣工。沈本高速公路高台子新出口完成工程量的50%。本桓铁路也开始前期工作。电网、通讯网建设完成投资20.4亿元。本溪热电项目可研报告已上报国家审批。风电和煤矸石发电等项目正在推进前期工作。争取到国家拉动内需项目141个、资金总额5亿元。

加快生物医药产业发展，中国药都正在加速崛起。去年新签约入驻辽宁成大、大连美罗、天津天士力等项目52个，新增投资额69亿元。药业基地累计入驻项目122个，投资总额144亿元，达产后年可实现销售收入360亿元。项目开工建设62个，完成主体工程30个，竣工投产和试生产15个。93个药业项目得到了省科技厅的重点扶持。基础设施建设和城市功能配套也在加紧推进与完善。当年新增建设用地6平方公里，园区路网、供水、污水处理和供电、供气、供热等设施全部运行。生物医药研发中心投入使用。辽宁科技学院搬迁入驻。沈阳药科大学、本溪高中分校、药业博物馆、行政服务中心、星级酒店、公交中转场和新城中心医院等开工建设。中国医科大学本溪校区完成选址。30万平方米商住开发已经竣工。五家商业银行设立分支机构。开发区与主城区公交车正式开通。过去这一年，开发区一年四季都是塔吊林立、红旗招展，建设场面热火朝天。我们正在用实际行动，将本溪几代人追求的发展医药产业的梦想加快变为现实。

逆势招商成效显著，财税金融工作明显加强。在日、韩、新、港、台和“长三角”、“珠三角”等地组织开展了招商活动30余次，接触国内外客商600余人次。成功举办了“辽宁（本溪）第二届生物医药高新技术交易会”。全市实际利用外资2亿美元，增长77%。引进内资220亿元，增长201.8%。资金到位的千万元以上项目275个。财税部门强化依法征收，确保了财政收入目标的完成。全市金融机构贷款余额560亿元，当年新增200亿元，增长56%，创历史最高水平。兴业银行作为本溪历史上第一家股份制银行即将营业。第一家村镇银行挂牌运营。本溪城信社获准筹建商业银行。充分发挥了城投公司政府融资平台的作用。15亿元城市建设投资债券发行已上报国家审批。正在筹建的24家小额贷款公司已开业9家。新组建7家担保公司。本溪市的金融环境明显改善，贷款规模大幅度增加，金融运行质量显著提高，标志着本溪今后的发展前景将会更加充满活力。

加快县域经济发展，农村经济综合实力不断增强。本溪县和桓仁县的工业园区基本实现“五通一平”，入驻企业73户，实现销售收入59亿元。本溪县中药材种植新增4.5万亩，桓仁县酿酒葡萄新增2400亩。进一步规划了两个县城的发展，房地产开发完成95万平方米。两县生产总值和财政一般预算收入平均增幅分别高于全市5个和15个百分点，为全市保增长做出了贡献。认真落实各项惠农政策，为47万人发放粮食综合补贴和农机补贴6016万元。全市粮食产量26.3万吨。新建设施农业1.1万亩、畜禽标准化养殖小区170个。35个重点农业产业化项目进展顺利。规模以上龙头企业达到104个。农田水利建设有望再次获得省“大禹杯”。扶贫开发不断深入。农业保险开端良好。本溪农业农村经济正在加快向优质化、设施化和特色化方向迈进。

突出抓好旅游和商贸流通，服务业和城区经济发展步伐加快。成功举办了升格为国家级节庆活动的第七届中国?本溪国际枫叶节。本溪水洞创建5A级景区通过省级初评，并纳入国家世界自然遗产预备名录。在香港成功推介了20平方公里本溪水洞国际旅游度假区的主题概念招商品牌。全年旅游总收入150.2亿元，增长32.8%。本溪新玛特购物广场、永丰商业区改造等项目竣工投入使用。明山卧龙地区初步形成了汽车4S专营店集群。房地产开发全年完成投资46.5亿元，新开工302万平方米，竣工265万平方米，在建总面积705万平方米，商品房销售面积173万平方米、交易额54.1亿元，上述指标都是历史最高水平。四个城区的生产总值和地方财政一般预算收入平均增幅均在20%以上，成为全市保增长的重要力量。

（二）千方百计保民生，人民群众生活实现新改善

坚持把增加就业放在保民生首位。全市城镇实名制就业8.25万人。劳务输出2.5万人。零就业家庭继续保持动态为零。高校毕业生就业率90%。400名贫困家庭高校毕业生由政府全部进行了兜底安置就业。全市城镇登记失业率控制在4.5%以内。

继续完善社会保障体系。“五项保险”新增扩面21万人，各项民生保障标准继续高于或保持全省平均水平。18.2万名企业退休人员养老金人均月增107元，并做到按时足额发放。下调了城镇职工基本医保起付标准和个人自付比例，减少个人支出3200万元。解决了466户集体困难企业、2.5万名退休人员的基本医保问题。城镇居民基本医保参保率80%。新农合参合率99.4%，报销封顶线提高到4万元。城乡低保标准分别提高50元和300元。

时刻关注弱势困难群体。全年累计发放城乡低保金1.8亿元。投入资金7160万元，大规模帮扶救助20.6万户次。实施城乡特困居民医疗救助7.8万人次。慈善事业健康快速发展。落实“两免三补”等专项资金7367万元，受益学生13.4万人。为1209户低保及边缘户发放租赁补贴215万元。为农村贫困户建房730户。新建和改建8所农村中心敬老院及1所儿童福利院。

积极建设惠民利民工程。新建廉租住房1617套、7.4万平方米，廉租房实物配租和租赁补贴5037户。对250万平方米的老旧管网及热源进行了集中改造。冬季供暖做到了按期开栓、基本稳定供热。新铺设79个住宅区人行甬道和车行道11万平方米，维修整治街巷路、“摸黑路”34条，两项便民设施建设任务基本结束。

切实加强生态建设。全市春秋植树造林27万亩，大规模冬季绿化造林4.9万亩。创建国家森林城市和全国绿化模范城市工作通过国家初评。溪湖区被列为国家资源综合利用实验区，南芬区被列为国家可持续发展实验区。实施重点减排项目36项。4个污水处理厂及千金岭生活垃圾处理厂全部投入使用。年度减排二氧化硫1.32万吨、化学需氧量0.98万吨，万元GDP综合能耗和取新水同比均下降4.4%。全市环境空气质量二级（良）以上天数343天，城市环境综合考评晋升为全省第5位。

不断强化城市规划建设和管理。高标准编制了本溪新城的控制性详规和市政基础设施专项规划，并推进了基础设施建设。主城区改造了总长47公里的彩屯、溪湖和竖井等20条道路。城区绿化栽植树木7万株，新建改造绿地30万平方米。新增楼体亮化36栋。大力开展“城乡清洁工程”和爱国卫生清洁月活动，深入整治了城区30条主次干路两侧市容环境，主城区大街小巷以及铁路、公路沿线和城乡结合部环境卫生面貌焕然一新。

（三）集中精力保稳定，和谐本溪建设又有新进展

着力加强安全生产、信访稳控和社会治安管理。不间断地组织安全生产大检查，全市事故发生起数下降25.6%、死亡人数下降8.5%。扎实开展矛盾纠纷排查化解等活动，妥善处理和解决了一大批重点疑难信访案件，全市信访形势总体平稳。圆满完成了国庆安保工作任务，市公安局荣获全省国庆安保先进集体。本溪市的密切警民关系、治安巡逻防控和公安信息化建设三项工作走在全国前列。全市可防性刑事案件和治安案件分别下降64%和67%，现行命案破案率达到100%。社会治安秩序持续好转，人民群众的安全感、公正感和满意度进一步提高。

全面发展各项社会事业。在全省率先实现义务教育“双高普九”达标和全部完成农村寄宿制学校建设。高考总录取率88.9%。中职毕业生就业率92%。化工学校在全国技能大赛中获得“四连冠”。建成市传染病院二期和150个社区、乡村医疗卫生基础设施，全市新增医疗用房7万平方米。防控甲型H1N1流感、手足口病等突发公共卫生事件成效显著。文学艺术创作成果丰硕，《女人是座山》等三部文艺作品获得省“五个一工程”奖。设立并实施了首届哲学社会科学成果政府奖。本溪市博物馆正式开放。成功举办了市第九届运动会。本溪籍选手在第十一届全运会上夺得“两金一银一铜”的好成绩。本溪体育馆成功承办了8场高水平国际国内大型赛事。全国男排联赛辽宁队主场落户本溪。低生育水平持续稳定，人口自然增长率控制在0.35‰以内。食品药品监管和质量技术监督工作扎实有效。民族工作荣获全国民族团结进步模范市“五连冠”。争创第七轮“双拥模范城”通过省中期考核。新建农村社区服务中心74个。溪湖区等四家单位被评为全国和谐社区示范单位。统计、粮食、物价、外事、侨务、人防、气象、防震、档案、地方志、残疾人事业等都取得了新成绩。

（四）尽职尽责抓服务，政府自身建设得到新加强

市政府领导班子成员率先垂范，树立执政为民的理念，努力在勤政廉洁上高标准、严要求，坚持昂扬向上、亲历亲为、雷厉风行、动脑想事、弯腰干事，讲究依法行政、诚实守信、规范程序、提高效率、优质服务。市政府参加第二批学习实践科学发展观活动群众测评满意率95.7%。认真贯彻落实国务院《关于加强市县政府依法行政决定》，积极推进了以人为本行政执法新机制建设，政府科学决策水平不断提高，政务公开不断规范。提请市人大常委会制定地方法规3件，制定政府规章5件。自觉接受市人大、市政协的法律监督和民主监督，全年办理人大代表建议和政协提案966件，办复率100%、解决率68.1%。新一轮政府机构改革基本完成。市长公开电话和市长信箱分别受理市民投诉15533件（次）、网上信件3213件，办结率98.3%。不断强化行政监察和审计监督，建设用地、重点项目和政府采购均实行了公开挂牌招投标，做到了政府资金到哪里，审计监察就跟踪到哪里。

各位代表，回顾过去一年应对金融危机保增长的实践和本溪新的发展变化，至少说明了本溪市贯彻党中央、国务院和省委、省政府应对金融危机的各项决策部署态度是坚决的，所做的各项工作是到位的，采取的各项措施是及时的，否则就不会有过去一年这样好的成效；说明了我们抓住了金融危机这个机遇，变危机为机遇，为本溪的长远发展打下了基础，现在正在建设的高速公路、铁路、城际轨道等项目，现在发展的生物医药产业等，虽然三五年以后才能见到成效，但这些都为本溪长远发展、可持续发展奠定了坚实的基础，这更是本届政府应尽的责任；说明了全市上下在困难面前头脑是清醒的、思路是清晰的，工作是有潜力、有能力、有作为的，广大干部群众是团结的、是有战斗力的。一年多的时间，使我们充分感受了什么是危机，危机对我们这座城市有什么影响，我们应以什么样的状态和作风去应对危机，更使我们知道了如何将危机变成机遇。全市上下积累了经验、锻炼了队伍、坚定了信心、增加了激情、明晰了思路，这是最难能可贵的精神财富。只要我们一鼓作气、锲而不舍地抓下去，任何困难面前都会风雨无阻。

各位代表，过去的一年，我们经历了国内钢铁产品价格的大幅走低，面对了国际出口市场的急剧萎缩，克服了多年以来少有的严重旱灾，圆满完成了保增长、保民生、保稳定、促振兴的各项目标任务。这是省委、省政府和市委正确领导的结果，是市人大、市政协监督支持的结果，是全市各族人民团结一心、努力奋斗的结果。在此，我代表市政府，向辛勤工作在各行各业的全市人民，向给予政府工作大力支持的各级人大代表和政协委员、各民主党派、工商联、无党派人士、各人民团体和社会各界人士，向驻溪中省直单位和驻溪解放军、武警部队官兵表示衷心的感谢!

总结过去一年的工作，我们清醒地认识到，当前全市还面临许多问题：经济发展的结构性矛盾还很突出，钢铁深加工和旅游两个产业集群还没有大的进展，各县区工业园区的产业特色还不明显。全市的开放度还不高，利用外资项目少、规模小。两县和四区经济发展在全省的位次不够靠前，发展潜能还没有真正释放出来。就业和社会保障的压力在持续增加，部分城乡居民生活还比较困难，老工业基地积累下的矛盾逐渐显现，维护稳定的难度加大。政府职能还不能完全适应新形势、新任务要求等等。对此，我们有信心、有能力在新的一年里认真地加以改进和解决。

二、2010年主要工作任务

2010年是“十一五”规划的收尾之年，也是为“十二五”高起点开局的奠基之年。综观全局，国际金融危机的影响仍然存在，国内经济回升的动力仍然不足，谋求经济平稳较快发展的挑战与压力增大。全市上下既要增强必胜信心，更要增强忧患意识，全面贯彻落实十七届四中全会和省委十届九次全会、中央和省经济工作会议精神，全面贯彻落实市委十届八次全会精神，调结构、稳增长、惠民生、保稳定， 努力在新起点上高标准做好各项工作。

新的一年，市政府将立足当前，全力抓好“十一五”的总结收尾，落实好本溪经济社会发展的各项工作任务；还要谋划好长远，编制完成“十二五”规划，精心绘制好本届政府后三年乃至“十二五”全市经济社会发展的规划蓝图：我们要加大力度打造20平方公里的中国药都、20平方公里的钢铁深加工产业园区、20平方公里的本溪水洞国际旅游度假区，将本溪尽快发展升级为由多元产业支撑的现代城市;我们要加快做强做大县域经济和城区经济，全面实现“三年翻一番、六年翻两番”的奋斗目标;我们要通过不懈努力，抓投资、上项目、增后劲，使本溪尽快形成几个新的产业、新的城区和新的现代化的基础设施;我们要树立更高的奋斗志向，用三五年时间，使全市的生产总值达到1000亿元，固定资产投资达到1000亿元，贷款余额达到1000亿元，地方财政一般预算收入达到100亿元;我们要携手并肩，把本溪建设成为钢都、药都、枫叶之都，建设成为中国优秀旅游城市、国家森林城市、国家园林城市、国家卫生城市和全国环保模范城市。

谋求更好更快发展，实现预期目标，市政府工作要突出以下六个着力点：着眼于经济快速发展，必须集中精力解决好决定发展的土地、资金和项目三个关键问题；着眼于提升城市水平，必须把握好改造主城区、开发新城区和在沈阳与本溪的节点上脱离主城建新城三个发展阶段；着眼于调整产业结构，必须加快推进生物医药、钢铁深加工和旅游三个产业集群的建设；着眼于改善民生，必须切实抓好就业再就业、社会保障和棚户区改造三大工程；着眼于构建和谐社会，必须时刻抓好社会治安、信访稳控和安全生产三项重点工作；着眼于提高政府效率，必须以务实、勤奋、廉洁三条要求为标准，不断加强政府公务员队伍勤政廉政建设。我们只有将这“六个三”坚持不懈地把握住，不断地有新突破，不断地有新作为，本溪经济社会发展才能一年比一年快，效益才能一年比一年好，变化才能一年比一年大。

2010年全市经济社会发展的主要预期目标是：地区生产总值增长15%，规模以上工业增加值增长20%，地方财政一般预算收入增长13%，固定资产投资增长40%，社会消费品零售总额增长18%，实际利用外资增长50%，出口创汇增长30%，引进内资增长20%，城镇居民人均可支配收入增长10%，农民人均纯收入增长12%。在实际工作中，我们要坚持增量调结构，在调整中扩增量，努力实现主要经济指标增幅不低于振兴以来平均水平，不低于全省各市平均水平。

今年要重点做好以下工作：

第一，以工业为主体组织好经济运行，确保全市财税收入持续增长。全力保障重点企业、重大项目的生产要素配置。保持工业用电量的持续增长。积极促进本钢、北钢等重点骨干企业实现稳产、增产。继续解决好国企改革的遗留问题。引导和支持中小企业、民营企业调整产品结构，推动企业自主创新发展。抓好400个技术改造项目。推进并购海外科技型企业和引进海外研发团队工作。全市规模以上工业企业户数要达到750户。进一步加强银企对接和融资服务，支持和鼓励各金融机构加大信贷投入。在发行好15亿元城投债券的基础上，支持中小企业充分运用好集合债券、短期融资债券和中期票据等金融产品。推进本溪矿冶、聚丰公司和本溪冶炼等企业上市。力争再引进一两家股份制商业银行。本溪市商业银行要正式挂牌营业。全市小额贷款公司和村镇银行总数要达到30家。全市金融机构要新增贷款140亿元，贷款余额达到700亿元。坚持依法治税和社会综合治税，严格审核管理和大力压缩财政支出，确保财政收支平衡。

第二，全力抓好固定资产投资，全年投资总额要实现500亿元。全力以赴、时刻不停地滚动推进总投资5000亿元的1000个固定资产投资项目，重点抓好今年续建和新建的总投资1900亿元的630个项目。沈本城际轨道项目土建要全面开工。桓仁“两高一铁”要完成路基改造等主体工程。沈本产业大道收尾和沈本高速公路高台子新出口要全部竣工。抓紧推进本桓铁路和本桓高速公路前期工作，尽快在本溪市区和桓仁县之间形成高效便捷的交通走廊。本溪热电联产、桓仁抽水蓄能电站以及国家电网1000千伏特高压输变电工程要争取开工。要加快推进本钢三冷轧、引观入本等重点项目建设。

第三，实施建设本溪和沈阳之间的产业带和城市带，加快沈本一体化进程。尽快编制完成《沈本城际连接带规划》。今年要把中国药都沿沈本产业大道两侧向沈阳方向再扩大10平方公里，延伸到本溪和沈阳的交界线。要以医疗器械产业招商建设为重点，同步实施供电、给排水等基础设施建设。建成一座交通景观大桥。完成30公里的路网和20公里的生态水系建设。新建20万平方米的生态公园、12万平方米的城市主题公园和3万平方米的城区中心广场。推进土地置换，建设10万平方米的集中安置回迁住宅。积极推进二级火车站、二级客运站和新城体育中心的规划建设。开通沈本新城到沈阳的公交线路。药业博物馆、行政服务中心、移动通讯大厦、公安消防综合楼以及星级酒店等要在年底前完成主体工程。同时，还要规划和推进从沈本新城向本溪方向延伸的城镇化和产业带建设。

第四，大力发展以生物医药为主的新兴产业，坚定不移地推进中国药都建设。中国药都的“药”是产业，“都”是城市，两者是一个有机整体，必须齐头并进。要以囊括四海的气魄和胸怀、坚韧不拔的斗志和干劲，坚定200个项目聚集、千亿元产业规模的目标不动摇、不松劲。进一步加大力度、加快进度，不仅抓招商、抓建设，更要抓产出，提高竞争力、知名度和影响力。要重点瞄准南京医药、石药集团、敖东制药等国内外大型药业企业，紧紧围绕医疗器械、疫苗生产及研发生产外包等抓好专项招商。完成300个以上药品品种的划转和注册。到年末力争实现60个项目投产运营、60个项目在建、60个项目签约入驻并做前期设计、销售收入60亿元。再新增4平方公里建设用地，完成征地拆迁和配套基础设施建设。新城中心医院要基本建成。支持中国医大和沈阳药大本溪校区加快建设步伐。本溪高中分校要竣工使用，石桥子中小学要划归市实验中学和小学。全年中国药都固定资产投资要达到100亿元。聘任一批全国医药行业的知名专家作为政府顾问，指导、帮助和谋划本溪药业发展。加大药业自主研发力度，提高中国药都的核心竞争力。进一步加强对药业企业生产许可、认证和注册等方面的服务。积极申报省高新技术产业开发区、国家生物医药科技产业基地和国家医疗器械科技产业基地。

第五，充分依托两大钢铁企业，加快推进20平方公里钢铁深加工产业园区建设。打通本钢和北钢之间的交通连接。充实完善20平方公里钢铁深加工产业园区的发展规划。积极推进大台沟铁矿开发。依托本钢、北钢两大企业，重点瞄准装备制造、成套设备的配套产品加工，延长钢铁产业链。园区产业规模要达到500亿元以上。抽调精干力量、组成专门队伍，下大气力推进产业园区的融资、建设用地储备、基础设施建设以及主题概念招商。今年要在起步区内建成园区环路以及供电、供水等配套基础设施，确保招商入驻项目50个以上。

第六，着力提升旅游业，加快推进20平方公里本溪水洞国际旅游度假区开发建设。按照“水洞、温泉和枫叶”三位一体的发展思路，加快理顺管理体制和相关利益关系，高标准地编制和实施本溪水洞国际旅游度假区发展规划。完成水洞到温泉寺5公里道路改造工程，新建一座跨河大桥。推进温泉寺核心区供电、给排水、供暖等配套设施建设。完成10公里太子河水系治理工程。要在规划区内道路两侧、山上山下、山地河边大规模营造枫树带、枫叶园，种植枫树10万株以上。制定招商政策，推进主题招商和专题招商，力争在融资、温泉开发、温泉寺国际会议中心建设等方面取得实质进展。同时，本溪水洞景区要确保实现创建国家5A级旅游景区目标，并积极申报世界自然遗产。要继续推进桓仁八卦城恢复、关门山铁刹山资源整合等重点项目建设。切实加大旅游宣传促销和市场开拓工作力度，全年接待各类游客人数及旅游总收入均要增长30%以上。

第七，落实城乡统筹，促进县域经济大发展。积极争取将本溪作为沈阳经济区统筹城乡发展综合配套改革试点市，将本溪县作为全省城乡统筹试点县，进一步推进新农村建设。本溪县和桓仁县要明确和突出工业园区的产业定位与特色，加大招商力度，推进项目入驻和产业集聚，尽快形成100亿元的产业规模。两县要加快做大“一县一品”的农业产业规模。本溪县新种植中药材5万亩以上，桓仁县新发展酿酒葡萄基地8000亩。同时，着力推进汇源果汁加工、王朝酒庄等农业产业化重点项目建设。要高起点地推进本溪、桓仁两个县城的中等城市规划建设，今年两县房地产开发都要达到50万平方米以上。到年底，两县主要经济指标要确保实现比2007年翻一番。大力推进农业产业结构调整，全市要新发展耕地及林下中药材11万亩、山野菜3.7万亩、干果经济林9万亩。建设市级以上现代农业示范基地37个、设施农业1万亩、省级畜禽标准化养殖小区150个。加强粮油储备管理。进一步深化集体林产权配套制度改革。深入抓好农村能源、环境保护、农田水利、河道治理等基础建设。

第八，以服务业和园区建设为重点，加快推进城区经济发展。进一步活跃和繁荣城乡市场，拉动居民消费较快增长。着力推进大商集团投资15亿元的地工路商业地产、总投资3亿元的胜利路三角地改造以及1.5万平方米的新城华联超市等项目。积极引进知名商业企业进驻大峪地下人防工程。支持本钢国贸、溪源车业、平安车业等20多家物流企业发展。加快打造明山卧龙地区的汽车4S店产业集群和溪湖彩北商贸物流集中区。尽快引进家乐福、新世界等高档商业品牌。推进大河市场资源整合。切实抓好平山区钢铁深加工、明山区汽车零部件、溪湖区新型建材和南芬区方解石新材料四个工业园区建设。今年四个城区的五大经济指标均要增长25%以上，在全省56个城区的排位要努力前移3位。

第九，进一步加大招商和安商工作力度，使资金、技术和人才大量涌入本溪。突出钢铁深加工、生物医药和旅游等产业集群发展的概念招商和专题招商，在积极引进生产加工型项目的同时，力争在引进现代服务业、房地产业及公益事业项目方面实现突破。帮助和推进有条件的企业实现境外上市。认真筹办第三届药交会并争取上升为国家级活动。在巩固发展好传统招商市场的同时，积极开辟欧美、东南亚和京津唐等新市场。全年组织招商活动50次以上。全市实际利用外资要力争实现5亿美元，引进内资要实现260亿元以上。进一步优化安商的服务环境，确保项目和人才招得来、留得住、发展好。继续鼓励和支持企业调整产品结构，扩大出口。全市出口创汇额要达到8亿美元。

第十，继续改善生态环境，提升本溪城乡建设管理水平。实施好本钢烧结机脱硫、本溪水泥厂环保搬迁等14个节能减排重点项目。在有效控制增量基础上，确保实现减排二氧化硫0.46万吨、化学需氧量0.91万吨。万元生产总值综合能耗下降4.4%。坚决实现20万亩绿化造林三年任务两年完成，全年造林10万亩以上，今年要确保荣获“国家森林城市”和“全国绿化模范城市”。完成市、县、乡土地利用总体规划，促进城乡建设布局结构调整。改造滨河南路、地工路、峪明路等6条市政道路。开工建设北地跨线桥。基本建成育龙路西段。继续加大房地产开发力度，全市要新开工400万平方米，竣工300万平方米，完成投资50亿元。继续下大力气提升城区的绿化美化亮化水平，新栽各种树木7万株。积极探索加强城市停车设施的建设和管理。以创建国家卫生城市为目标，重点整治60个村庄环境，为全市人民创造“天天干净、处处清洁”的人居环境。

十一，竭尽全力改善民生办实事，让改革开放和经济发展的成果更多地惠及本溪人民。为老百姓干活、为老百姓做事、为老百姓谋利益，这是本溪市政府的根本职责。今年要集中精力办好十件实事：一是城镇实名制就业8万人，稳定就业率70%以上；城镇登记失业率控制在5%以内。二是全市18.2万名退休人员养老金月人均再增加120元左右，并做到按时足额发放。三是推进桓仁县新型农村社会养老保险试点工作。四是提高医疗保障水平。城镇职工医保最高支付限额提高到6.2万元，城镇居民医保财政补助年人均标准提高到120元，新农合财政年人均补助标准提高到120元，最高支付限额提高到4.5万元。五是研究制定困难企业在职职工的医保政策，逐步解决其医疗保障问题。六是失业保险金待遇月人均增加120元以上。七是适时提高城市低保分类救助和农村低保标准，资助所有的城市低保及边缘户参加城镇居民基本医疗保险。八是完成250万平方米的老旧管网及热源改造任务。九是推进棚户区改造工程。新开工建设廉租房1万平方米，完成实物配租和租赁补贴5400户。十是落实优待抚恤补助标准自然增长机制，将标准提高15%-30%。在实际工作中，市政府还将扎实做好涉及民生民利的其他各项工作,让广大群众实实在在感受到每年生活都有新改善，一年更比一年好。

十二，协调发展社会事业，毫不松懈地抓好治安、信访和安全生产。积极促进教育公平，推动义务教育均衡发展、普通高中教育优质发展、职业教育特色发展。继续落实好“两免三补”、中职扶困助学等政策。完成校舍安全工程年度计划。深化医药卫生体制改革，落实9类国家基本公共卫生服务项目。整合城市医疗资源，构建二级医疗架构。续建和开工建设4座医疗卫生机构。新改扩建20个社区（村）卫生服务中心（卫生室）。积极稳妥地推进艺术团体、书店等文化体制改革。加快建设钢铁和水利工程博物馆。改造市文化艺术中心。规划建设本溪新城群众文化艺术中心和市档案馆。广泛开展全民健身运动，开发培育竞技体育项目和人才，在第十一届省运会上争创佳绩。开通高清电视和手持电视频道。深化人口和计划生育综合改革，人口自然增长率控制在1.53‰以内。认真开展全国第六次人口普查。重视发展老龄、红十字、社会福利和慈善事业。积极开展残疾人社会保障和社会服务体系建设。切实保障妇女和未成年人权益。促进外事、侨务和对台工作健康发展。加强物价、供销、气象、史志等各项工作。农村社区建设要实现全覆盖。巩固发展国防动员、民兵预备役和人防工作，积极争创全国双拥模范城“七连冠”。继续争创民族团结进步模范市。做好“五五”普法依法治市规划的检查验收。抓好新一轮综合治理先进市争创工作。继续加大社会治安巡逻防控和打击刑事犯罪工作力度，进一步推进公安“三大建设工程”。建设市、县（区）“一站式”信访接待大厅，切实抓好矛盾纠纷化解和信访稳控。继续防控好甲型H1N1流感等重点传染病。扎实做好森林防火、畜禽防疫和防汛抗旱。不间断地开展大检查和重点行业专项整治，确保安全生产形势稳定。

各位代表，新的形势、新的任务，对做好政府工作提出了更高要求。面对人民的期盼和重托，我们要把全部精力用在调结构、上项目、促发展上，用在抓节能减排、生态建设和为民造福上，用在化解各种矛盾、治理各种隐患，保安全、保稳定、促和谐上，始终对人民负责、受人民监督、让人民满意。对群众要有深爱之情。要从群众的呼声、意愿中把握推动发展的着力点，从群众的智慧、力量中汲取应对困难的好办法，从群众的实践、经验中寻找推动工作的突破口，多出一些亲民之策、为民之举，多办一些顺民意、解民忧、增民利的实事，真正为群众排忧解难、雪中送炭。对学习要有致用之道。少搞应酬多学习，挤出时间多看书，用学而不厌的毅力、学而乐思的精神，学出真本领，学到真东西。注重学以致用，把学习的成效真正体现到推动本溪发展振兴的工作实践中去，不断提高干事创业和应对驾驭复杂局面的能力。对工作要有守土之责。政府全体公务员每时每刻都要顶住压力、负起责任、化解矛盾、解决问题。各级领导干部特别是主要领导同志要带头昂扬向上，充满工作激情，把精力集中在想干事上，本领体现在会干事上，目标锁定在干成事上。把所有的目标工程化、具体化，对主要工作和重点项目要亲历亲为、事必躬亲、事不过夜，用工作提速、效率提高、形象提升的实际行动，营造本溪更加宽松、优越和便利的发展环境。对监督要有自觉之态。主动接受市人大的依法监督和市政协的民主监督，坚持定期报告和通报工作情况。主动听取民主党派、工商联、无党派人士和各人民团体的意见。认真办理人大代表建议和政协提案。重视新闻舆论和社会公众监督，支持监察、审计部门依法行政监督。加强法治政府建设，大力推进政务公开。对法纪要有敬畏之心。严格遵守廉洁自律的各项规定，远离诱惑，淡泊名利。严格落实“一岗双责”的党风廉政建设责任制，把廉政要求落实到每一个权力行使环节，切实做到既勤政又廉政,既干事又干净,树立政府为民、务实、清廉的良好形象。

各位代表，过去的一年，我们经受住了国际金融危机的严峻考验。新的一年，全市人民一定能够战胜各种困难，取得改革开放和现代化建设的新胜利。让我们在省委、省政府和中共本溪市委的正确领导下，深入学习实践科学发展观，满怀激情抓发展，千方百计促振兴，抓住新机遇、建设新本溪，为实现本溪经济社会发展的各项目标而努力奋斗！
